# Perceived subjective support is associated with self-efficacy in older adults early after intestinal stoma surgery: a cross-sectional study with FDR correction

**DOI:** 10.3389/fpsyg.2026.1850651

**Published:** 2026-08-07

**Authors:** Lijiao Wei, Lan Li, Sen Zhang, Ming Qiu, Xijie Hou, Ruili Wei, Yunhong Lu

**Affiliations:** 1 Department of Surgery, The First Affiliated Hospital of Guangxi Medical University, Nanning, China; 2 School of Nursing, Guangxi Medical University, Nanning, China

**Keywords:** aged, cross-sectional studies, health psychology, intestinal stoma, self-efficacy, subjective support

## Abstract

**Introduction:**

The early postoperative period after intestinal stoma surgery involves psychosocial adaptation for older adults. Self-efficacy is related to stoma care, social participation, and daily self-management. Social support includes objective support, subjective support, and support utilization. The cross-sectional association between these dimensions and self-efficacy remains insufficiently described in older adults early after intestinal stoma surgery.

**Methods:**

This single-center cross-sectional study recruited 162 consecutively sampled older patients (≥60 years) at 1–3 months after their first intestinal stoma surgery. The Social Support Rating Scale (SSRS) assessed total social support, objective support, subjective support, and support utilization. The Stoma Self-Efficacy Scale (SSES) assessed total self-efficacy, stoma care self-efficacy, and social self-efficacy. Pearson correlation analyses were corrected using the Benjamini-Hochberg false discovery rate (FDR) procedure across 36 tests. Multiple linear regression models used available covariates. Residual diagnostics, HC3 robust standard errors, and formal interaction tests were performed.

**Results:**

Total social support was not correlated with total self-efficacy after FDR correction (*r* = 0.095, *p* = 0.227, *q* = 0.584). Subjective support was positively correlated with total self-efficacy (*r* = 0.316, *p* < 0.001, *q* = 0.002) and social self-efficacy (*r* = 0.255, *p* = 0.001, *q* = 0.019). In available-covariate regression models, subjective support was associated with SSES-based total self-efficacy (*B* = 1.790, *β* = 0.438, *p* < 0.001), stoma care self-efficacy (*B* = 0.819, *β* = 0.281, *p* = 0.001), and social self-efficacy (*B* = 0.658, *β* = 0.329, *p* < 0.001). The corresponding *R*^2^ values were 0.159, 0.069, and 0.095; these SSES-based results were interpreted as exploratory because of the scale’s limited internal consistency in this cohort.

**Conclusion:**

Subjective support was associated with SSES-based self-efficacy scores in older adults early after intestinal stoma surgery. Given the limited internal consistency of the SSES in this cohort, these findings should be interpreted cautiously as exploratory associations rather than definitive evidence.

## Graphical abstract

**Figure:**
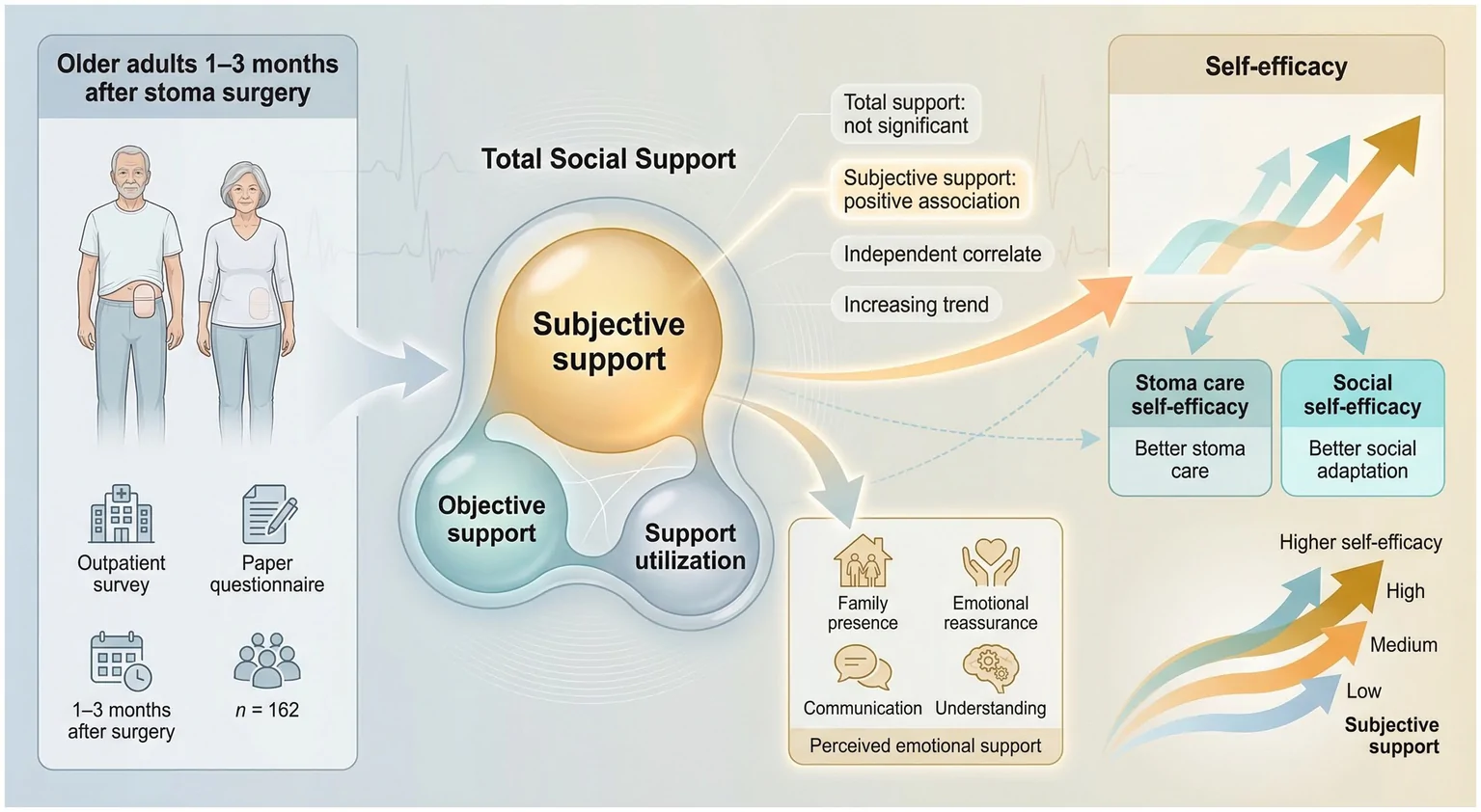
Subjective support and self-efficacy in older adults early after intestinal stoma surgery.

## Introduction

Intestinal stoma is a common surgical intervention for colorectal cancer and other intestinal diseases. With the persistent global burden of colorectal cancer, the population of patients undergoing intestinal stoma surgery and living with an intestinal stoma long-term continues to increase (Bray et al., 2024; Morgan et al., 2023). Postoperative patients face altered defecation patterns, body image changes, appliance management, and shifts in social roles (Tan et al., 2024; Duluklu and Celik, 2024). During the early postoperative period, patients relearn stoma care, dietary regulation, and daily activity management, with physiological, psychological, and social adaptation occurring in parallel (Ko et al., 2023; Martin-Gil et al., 2024). These issues are relevant for older patients, whose recovery may be affected by physical decline, chronic conditions, and reduced self-care capacity (Leite Junior et al., 2025; Wei et al., 2025).

Self-efficacy refers to confidence in performing specific health behaviors and is related to chronic disease management and rehabilitation (Bozkul et al., 2024; Xu et al., 2024). In patients with intestinal stoma, self-efficacy includes confidence in stoma care, complication management, outdoor activities, and social interaction (Aker and Karazeybek, 2025; Ozden and Kilic, 2023). Higher self-efficacy has been associated with better stoma adaptation, self-management, and quality of life (Bozkul et al., 2024; Xu et al., 2024). Video education, multimedia guidance, and continuous care have been reported to improve self-efficacy and stoma adaptation in intervention studies (Ozkaya and Harputlu, 2024; Ko et al., 2023; Huang et al., 2021).

Social support includes objective support, subjective support, and support utilization. Studies in patients with intestinal stoma have reported associations between perceived social support, self-efficacy, self-management, and psychosocial adaptation (Aker and Karazeybek, 2025; Xu et al., 2024). Patients with intestinal stoma also report support needs related to emotional adjustment, information, professional care, body image, social participation, and sexual adaptation (Wang et al., 2024; Fourie et al., 2025; Redeker et al., 2025). In older adults after intestinal stoma surgery, perceived support and objectively available support may show different correlations with self-efficacy (Wei et al., 2025; Aker and Karazeybek, 2025).

Research on quality of life, self-management, and psychological adaptation in patients with intestinal stoma has increased, but data on older adults during the early postoperative period remain limited (Leite Junior et al., 2025; Wei et al., 2025). Previous studies often used total social support scores as the main exposure, with fewer analyses of subjective support, objective support, and support utilization as separate dimensions (Aker and Karazeybek, 2025; Xu et al., 2024). This study examined social support and self-efficacy in older adults at 1–3 months after first intestinal stoma surgery and evaluated cross-sectional associations between social support dimensions and self-efficacy outcomes.

## Materials and methods

### Study participants

A single-center cross-sectional survey was conducted in the outpatient department of a tertiary hospital in Nanning, Guangxi, from May to December 2024. Participants were older adults who had undergone first intestinal stoma surgery and were 1–3 months postoperative. The inclusion criteria were as follows: (1) age ≥60 years; (2) first-time intestinal stoma surgery with a postoperative duration of 1–3 months; and (3) adequate literacy and communication skills to complete the questionnaire. The exclusion criteria were as follows: (1) mental illness or cognitive impairment; (2) comorbidity with other malignancies; (3) severe postoperative complications; and (4) critical illness with physical frailty. Based on empirical sample size estimation for multiple linear regression and potential questionnaire attrition, the final sample size was established at 162 (Supplementary Figure S1). The analyzable dataset included age, sex, SSRS scores, and SSES scores. Stoma type, primary diagnosis, postoperative complications, time since surgery, educational level, marital status, living situation, number of comorbidities, psychological distress, functional dependence, and prior self-care experience were not systematically available for covariate adjustment.

This study was approved by the Medical Ethics Committee of our hospital. Written informed consent was obtained from all participants. The principles of voluntary participation and confidentiality were followed.

### Survey instruments

The Social Support Rating Scale (SSRS), developed by Xiao Shuiyuan, was used to assess social support. This self-report scale comprises 10 items across three dimensions: objective support (items 2, 6, and 7), subjective support (items 1, 3, 4, and 5), and support utilization (items 8, 9, and 10). Most items use a 4-point Likert scale, whereas the number of selected support sources scores items 5 through 7. The total score is the sum of the three dimensions, with higher scores representing higher social support. Previous studies have reported reliability, validity, and structural stability for this scale (Zhang et al., 2024).

The Stoma Self-Efficacy Scale (SSES) was used to assess self-efficacy. Originally proposed by Bekkers et al. (1996) this self-report scale assesses stoma care and social functioning in patients with intestinal stoma. In this study, the 28-item version was adopted, comprising two dimensions (stoma care self-efficacy and social self-efficacy) and six individual items. The scale uses a 5-point Likert scale, where 1 indicates “no confidence” and 5 indicates “very confident.” The total score is obtained by summing individual items, ranging from 28 to 140, with higher scores representing higher self-efficacy. Previous validation work has reported much higher internal consistency than that observed in the present cohort, including original subscale Cronbach’s alpha values of 0.94 for stoma-care self-efficacy and 0.95 for social self-efficacy, Turkish validation values of 0.95 for the total scale, 0.92 for stoma-care self-efficacy, and 0.93 for social self-efficacy, and Chinese validation values cited in the validation literature of 0.97 for stoma-care self-efficacy and 0.89 for social self-efficacy (Karacay et al., 2020). A recent Czech validation study of the 28-item SSES also reported high reliability for the total scale and strong reliability across extracted factors, with a three-factor structure including stoma care, social, and burden self-efficacy domains (Machalkova et al., 2025). Because both SSRS and SSES are self-report measures, common method variance, response bias, and social desirability bias were considered in interpretation.

### Data collection methods

Data collection was conducted by two uniformly trained stoma specialist nurses. Paper-based questionnaires were distributed to eligible older adults with intestinal stoma. Investigators used standardized instructions to explain the survey purpose and completion method. Participants completed the questionnaires independently within approximately 30 min. A total of 216 patients were assessed for eligibility, 46 were excluded before questionnaire distribution, 170 questionnaires were distributed, and 170 were retrieved. Eight questionnaires were excluded because missing items exceeded 20% of the questionnaire content or involved multiple key scale items. A total of 162 valid questionnaires were included in the final analysis, with an effective response rate of 95.2% among distributed questionnaires (Supplementary Figure S1).

### Statistical methods

Statistical analyses were performed using SPSS 26.0 software. Data underwent integrity checks and logical verification. Quantitative data were subjected to normality and homogeneity-of-variance tests. Data conforming to a normal distribution were expressed as mean ± standard deviation. Comparisons between two groups used Welch’s *t-*test, and comparisons among multiple groups used one-way analysis of variance (ANOVA). Data not conforming to a normal distribution were presented as median (interquartile range), and group comparisons were performed using the Mann–Whitney *U* test or the Kruskal-Wallis *H* test. Categorical data were presented as frequencies and percentages, and group comparisons used the chi-square test or Fisher’s exact test.

Internal consistency was assessed for the SSRS and SSES items using Cronbach’s *α*. For the SSES, additional reliability diagnostics were summarized in Supplementary Table S4, including mean and median inter-item correlations, corrected item-total correlation ranges, alpha-if-item-deleted ranges, and maximum floor/ceiling effects. Pearson correlation analysis was used to evaluate associations between social support dimensions and self-efficacy outcomes. The Benjamini-Hochberg FDR procedure was applied to the 36 correlation tests shown in Table 1. Raw *p-*values and FDR-adjusted *q* values were reported. Statistical significance for the correlation analysis was interpreted using *q* < 0.05.

**Table 1:** Correlations between social support and SSES-based self-efficacy outcomes.

Predictor	Outcome	*r*	*p-value*	*q* value
Total social support	Total self-efficacy	0.095	0.227	0.584
Total social support	Stoma care self-efficacy	0.053	0.501	0.838
Total social support	Social self-efficacy	0.115	0.145	0.452
Total social support	Diet selection efficacy	−0.135	0.087	0.355
Total social support	Confidence in sexual activity	0.110	0.163	0.452
Total social support	Confidence in sexual satisfaction	0.031	0.700	0.840
Total social support	Confidence in heavy labor	0.052	0.510	0.838
Total social support	Confidence in maintaining vitality	−0.036	0.645	0.838
Total social support	Confidence in stoma self-care	0.051	0.518	0.838
Subjective support	Total self-efficacy	0.316	<0.001	0.002
Subjective support	Stoma care self-efficacy	0.203	0.009	0.113
Subjective support	Social self-efficacy	0.255	0.001	0.019
Subjective support	Diet selection efficacy	0.014	0.857	0.892
Subjective support	Confidence in sexual activity	0.127	0.108	0.387
Subjective support	Confidence in sexual satisfaction	0.013	0.868	0.892
Subjective support	Confidence in heavy labor	0.189	0.016	0.142
Subjective support	Confidence in maintaining vitality	0.076	0.339	0.814
Subjective support	Confidence in stoma self-care	0.134	0.089	0.355
Objective support	Total self-efficacy	−0.042	0.594	0.838
Objective support	Stoma care self-efficacy	−0.065	0.409	0.838
Objective support	Social self-efficacy	0.041	0.606	0.838
Objective support	Diet selection efficacy	−0.174	0.027	0.159
Objective support	Confidence in sexual activity	0.036	0.645	0.838
Objective support	Confidence in sexual satisfaction	0.036	0.652	0.838
Objective support	Confidence in heavy labor	−0.037	0.643	0.838
Objective support	Confidence in maintaining vitality	−0.025	0.757	0.852
Objective support	Confidence in stoma self-care	0.024	0.758	0.852
Support utilization	Total self-efficacy	−0.061	0.437	0.838
Support utilization	Stoma care self-efficacy	0.003	0.974	0.974
Support utilization	Social self-efficacy	−0.060	0.449	0.838
Support utilization	Diet selection efficacy	−0.146	0.064	0.327
Support utilization	Confidence in sexual activity	0.113	0.154	0.452
Support utilization	Confidence in sexual satisfaction	0.016	0.835	0.892
Support utilization	Confidence in heavy labor	−0.033	0.680	0.840
Support utilization	Confidence in maintaining vitality	−0.181	0.021	0.150
Support utilization	Confidence in stoma self-care	−0.067	0.395	0.838

Pearson correlation coefficients are reported. *q* values were obtained using the Benjamini–Hochberg false discovery rate correction across the 36 correlation tests displayed in this table.

Before calculating total and subscale scores for the Stoma Self-Efficacy Scale, all item responses were rechecked against the original scoring instructions to verify coding direction and score aggregation. Cronbach’s alpha was then recalculated from the item-level data for the total SSES, the stoma-care self-efficacy dimension, and the social self-efficacy dimension. Because the resulting coefficients were low in this sample and were markedly lower than values reported in previous validation studies, the SSES was retained only as a stoma-specific exploratory outcome measure. Subsequent analyses involving SSES outcomes were therefore interpreted as reliability-limited associations rather than definitive evidence based on a psychometrically robust primary outcome.

Separate multiple linear regression models were established with total self-efficacy, stoma care self-efficacy, and social self-efficacy as dependent variables. The available-covariate models included age, sex, subjective support, objective support, and support utilization. Total social support and its component dimensions were not included in the same model. Regression coefficients, standardized regression coefficients, 95% confidence intervals (CIs), *p-*values, and variance inflation factors (VIFs) were reported. Model explanatory power was reported using *R*^2^ and adjusted *R*^2^. Residual normality, heteroscedasticity, residual autocorrelation, multicollinearity, and influential observations were evaluated using Shapiro–Wilk tests, Jarque-Bera tests, Breusch-Pagan tests, Durbin-Watson statistics, VIFs, and Cook’s distance. HC3 robust standard errors were used as a sensitivity analysis for regression estimates. Diagnostic and robust standard error results are reported in Supplementary Tables S1, S2.

Effect sizes were calculated for the univariate analysis using Cohen’s d for pairwise comparisons and η^2^ for multiple-group comparisons. Self-efficacy level was classified as low (≤65), moderate (66–102), and high (≥103) using the cutoffs applied in the current dataset. Because the high category contained one participant, main analyses used continuous self-efficacy scores. For sensitivity analysis, subjective support scores were grouped by sample tertiles. Tied values produced strata of ≤11, 12, and ≥13. A trend test was performed by treating the subjective support strata as an ordinal variable. Sex- and age-stratified analyses were exploratory. Formal interaction tests for subjective support by sex and age group were performed in full-sample models, with FDR correction across six interaction tests (Supplementary Table S3). All statistical tests were two-sided. Unless otherwise specified, *p* < 0.05 was considered statistically significant.

## Results

### General characteristics of the study subjects

This study included 162 older adults with intestinal stoma in the early postoperative period, including 114 males (70.4%) and 48 females (29.6%). Age ranged from 60 to 83 years, with a mean age of 65.81 ± 6.16 years. By age group, 126 patients (77.8%) were aged 60–69 years, 27 patients (16.7%) were aged 70–79 years, and 9 patients (5.6%) were aged ≥80 years. The sample was concentrated in males and the 60-69-year age group (Table 2).

**Table 2:** General characteristics of the study participants.

Variable	Category/statistic	n/Mean	%/SD
Sex	Male	114	70.4
Sex	Female	48	29.6
Age group	60–69 years	126	77.8
Age group	70–79 years	27	16.7
Age group	≥80 years	9	5.6
Age	Mean ± SD	65.81	6.16

Data are presented as *n* (%) unless otherwise indicated. Mean ± SD is shown for age.

### Reliability analysis of the scales

Cronbach’s *α* coefficients were 0.822 for the total SSRS, 0.894 for the subjective support dimension, 0.753 for the objective support dimension, and 0.778 for the support utilization dimension. After item-coding and score-aggregation verification, the recalculated Cronbach’s α coefficients for the SSES remained 0.378 for the total scale, 0.324 for the stoma-care self-efficacy dimension, and 0.019 for the social self-efficacy dimension. These results indicated limited internal consistency of the SSES in the present cohort rather than a scoring-direction error. Scale reliability results are shown in Table 3, and additional SSES diagnostics are summarized in Supplementary Table S4.

**Table 3:** Internal consistency of the SSRS and SSES.

Scale/Dimension	Items	Cronbach’s alpha
Total SSRS	10	0.822
Subjective support dimension	4	0.894
Objective support dimension	3	0.753
Support utilization dimension	3	0.778
Total SSES	28	0.378
Stoma care self-efficacy dimension	13	0.324
Social self-efficacy dimension	9	0.019

SSRS = Social Support Rating Scale; SSES = Stoma Self-Efficacy Scale. Cronbach’s α was calculated from item-level data after item-coding verification. The low Cronbach’s alpha coefficients for the SSES are substantially lower than those reported in previous validation studies and should be interpreted as evidence of limited internal consistency in the present sample; analyses using SSES outcomes should therefore be considered exploratory and reliability-limited.

Additional item-level diagnostics showed no missing SSES item cells (*N* = 162), very low mean inter-item correlations for the total scale and dimensions, and corrected item-total correlation ranges below the conventional 0.30 threshold. These supplemental diagnostics are summarized in Supplementary Table S4 and support treating SSES-based findings as exploratory and reliability-limited.

### Current status of social support and self-efficacy

The total social support score was 39.15 ± 4.57, with a range of 16 to 50. Objective support had the highest dimension score (19.86 ± 2.52), followed by subjective support (11.43 ± 1.99) and support utilization (7.85 ± 1.41) (Table 4).

**Table 4:** Descriptive statistics for social support and SSES-based self-efficacy measures.

Variable	Min	Max	Mean	SD
Subjective support	5	17	11.43	1.99
Objective support	8	26	19.86	2.52
Support utilization	3	11	7.85	1.41
Total social support	16	50	39.15	4.57
Stoma care self-efficacy	17	64	38.19	5.80
Social self-efficacy	16	38	24.91	3.99
Diet selection efficacy	1	5	3.10	0.80
Confidence in sexual activity	1	4	1.31	0.53
Confidence in sexual satisfaction	1	3	1.33	0.50
Confidence in heavy labor	1	5	1.93	0.65
Confidence in maintaining vitality	1	5	2.94	0.84
Confidence in stoma self-care	1	5	1.91	0.80
Total self-efficacy	52	123	75.61	8.14

Data are presented as mean ± SD with observed minimum and maximum values.

The total self-efficacy score was 75.61 ± 8.14, with a range of 52 to 123. Among the two primary dimensions, stoma care self-efficacy was 38.19 ± 5.80 and social self-efficacy was 24.91 ± 3.99. The six individual item scores were 3.10 ± 0.80 for dietary choice efficacy, 1.31 ± 0.53 for confidence in sexual activity, 1.33 ± 0.50 for confidence in sexual satisfaction, 1.93 ± 0.65 for confidence in heavy physical labor, 2.94 ± 0.84 for confidence in maintaining vitality, and 1.91 ± 0.80 for confidence in stoma self-care (Table 4).

### Stratified distribution of self-efficacy levels

**Figure 1:**
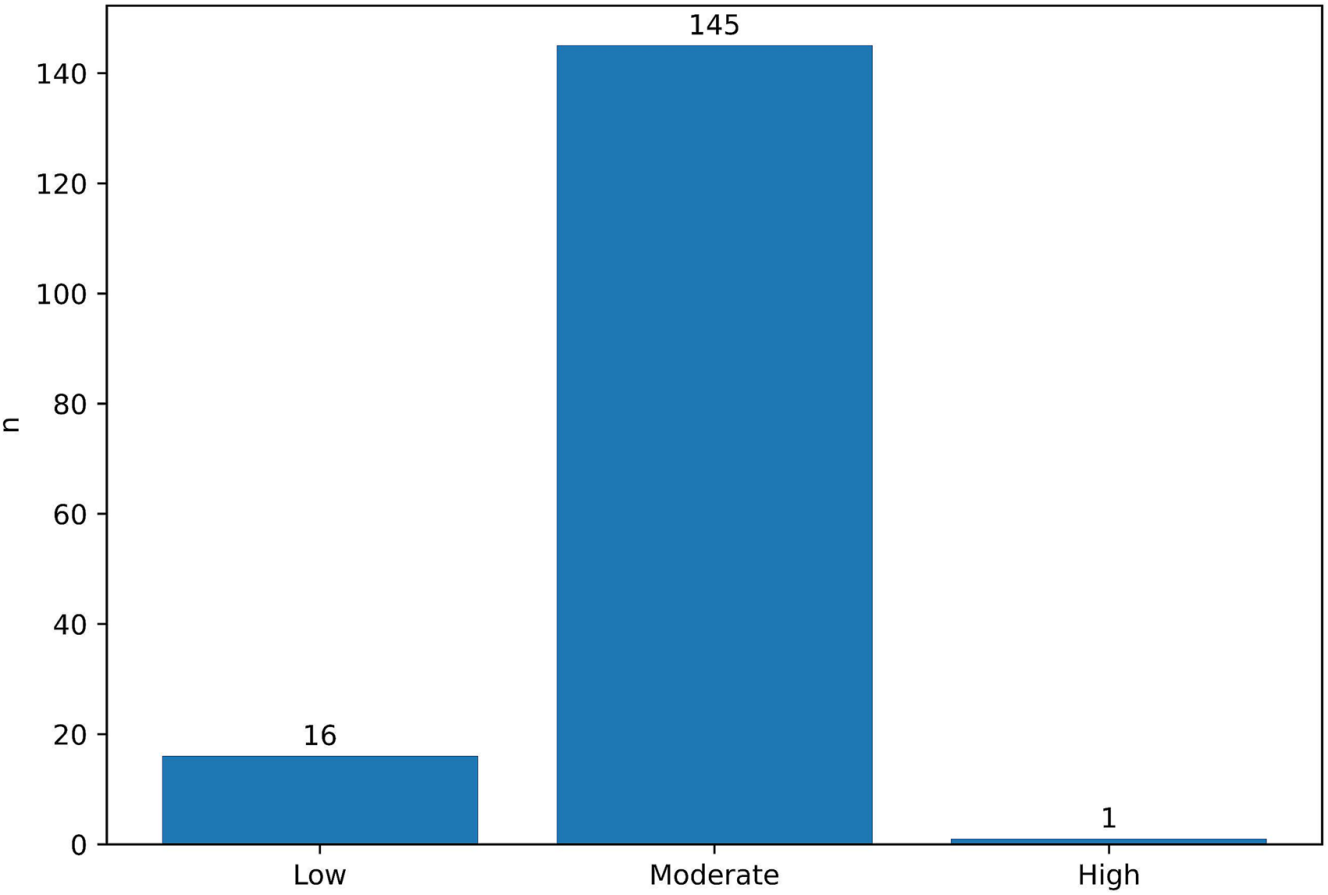
Stratified distribution of SSES-based self-efficacy levels among the study subjects.

Self-efficacy levels were classified as low (≤65), moderate (66–102), and high (≥103). There were 16 patients (9.9%) in the low category, 145 (89.5%) in the moderate category, and 1 (0.6%) in the high category. The high category was retained for descriptive reporting only (Table 5 and Figure 1).

**Table 5:** Distribution of SSES-based self-efficacy levels.

Self-efficacy level	*n*	%
Low	16	9.9
Moderate	145	89.5
High	1	0.6

Self-efficacy levels were classified using the cutoffs applied in the current dataset: low ≤65, moderate 66–102, and high ≥103. The high category contained only one participant and should be interpreted descriptively.

### Univariate analysis of differences in self-efficacy among patients with different characteristics

Sex-based comparisons showed no statistically significant differences in total self-efficacy, stoma care self-efficacy, or social self-efficacy. Total self-efficacy scores were 75.52 ± 8.90 in males and 75.83 ± 6.05 in females (*p* = 0.794, Cohen’s *d* = −0.039). Stoma care self-efficacy scores were 38.18 ± 6.10 and 38.21 ± 5.09 (*p* = 0.972, Cohen’s *d* = −0.006). Social self-efficacy scores were 24.92 ± 4.17 and 24.90 ± 3.56 (*p* = 0.969, Cohen’s *d* = 0.006) (Table 6).

**Table 6:** Exploratory univariate comparisons of SSES-based self-efficacy outcomes by sex and age group.

Outcome	Grouping variable	Group 1/Description	n1	Descriptive statistics 1	Group 2	n2	Descriptive statistics 2	Statistic type	Statistic value	*p-*value	Effect size type	Effect size
Total self-efficacy	Sex	Male	114	75.52 ± 8.90	Female	48	75.83 ± 6.05	t	−0.262	0.794	Cohen’s *d*	−0.039
Total self-efficacy	Age group	60–69/70–79/≥80 years	-	60–69 years: 75.98 ± 8.41; 70–79 years: 74.07 ± 7.33; ≥80 years: 75.00 ± 6.56				F	0.636	0.531	η^2^	0.008
Stoma care self-efficacy	Sex	Male	114	38.18 ± 6.10	Female	48	38.21 ± 5.09	t	−0.035	0.972	Cohen’s d	−0.006
Stoma care self-efficacy	Age group	60–69/70–79/≥80 years	-	60–69 years: 38.48 ± 5.92; 70–79 years: 37.19 ± 5.09; ≥80 years: 37.00 ± 6.34				F	0.754	0.472	η^2^	0.009
Social self-efficacy	Sex	Male	114	24.92 ± 4.17	Female	48	24.90 ± 3.56	t	0.039	0.969	Cohen’s d	0.006
Social self-efficacy	Age group	60–69/70–79/≥80 years	–	60–69 years: 24.98 ± 4.15; 70–79 years: 24.26 ± 3.28; ≥80 years: 26.00 ± 3.64				F	0.711	0.493	η^2^	0.009

For sex, Welch’s *t*-test was used; for age group, one-way ANOVA was used. Effect sizes are Cohen’s *d* for sex comparisons and η^2^ for age-group comparisons.

Total self-efficacy scores in the 60–69, 70–79, and ≥80-year age groups were 75.98 ± 8.41, 74.07 ± 7.33, and 75.00 ± 6.56, respectively (*p* = 0.531, η^2^ = 0.008). Stoma care self-efficacy scores were 38.48 ± 5.92, 37.19 ± 5.09, and 37.00 ± 6.34 (*p* = 0.472, η^2^ = 0.009). Social self-efficacy scores were 24.98 ± 4.15, 24.26 ± 3.28, and 26.00 ± 3.64 (*p* = 0.493, η^2^ = 0.009). The ≥80-year group included 9 patients (Table 6).

### Correlation analysis between social support and self-efficacy

**Figure 2:**
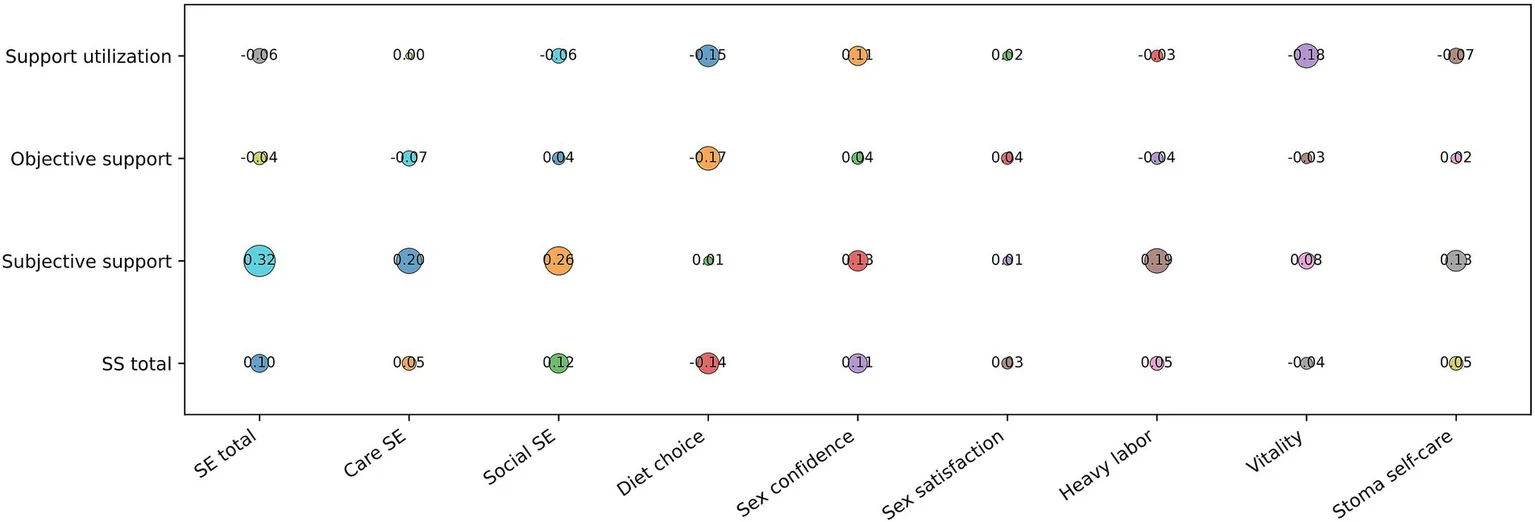
Bubble chart of correlations between social support dimensions and SSES-based self-efficacy outcomes.

Total social support was not significantly correlated with total self-efficacy after FDR correction (*r* = 0.095, *p* = 0.227, q = 0.584). It was also not significantly correlated with stoma care self-efficacy (*r* = 0.053, *p* = 0.501, *q* = 0.838) or social self-efficacy (*r* = 0.115, *p* = 0.145, *q* = 0.452) (Table 1 and Figure 2).

Subjective support was positively correlated with total self-efficacy (*r* = 0.316, *p* < 0.001, *q* = 0.002) and social self-efficacy (*r* = 0.255, *p* = 0.001, *q* = 0.019). The correlation between subjective support and stoma care self-efficacy had a raw *p-*value below 0.05 but did not remain significant after FDR correction (*r* = 0.203, *p* = 0.009, *q* = 0.113). Objective support and support utilization were not significantly correlated with the three main self-efficacy outcomes after FDR correction. Subjective support showed stronger associations with self-efficacy outcomes than the other social support dimensions in this sample (Table 1 and Figure 2).

For individual self-efficacy items, no correlation remained statistically significant after FDR correction. The correlations between objective support and diet selection efficacy (*r* = −0.174, *p* = 0.027, *q* = 0.159), support utilization and confidence in maintaining vitality (*r* = −0.181, *p* = 0.021, *q* = 0.150), and subjective support and confidence in heavy physical labor (r = 0.189, *p* = 0.016, q = 0.142) did not meet the *q* < 0.05 threshold (Table 1).

### Multivariate analysis of social support and self-efficacy

**Figure 3:**
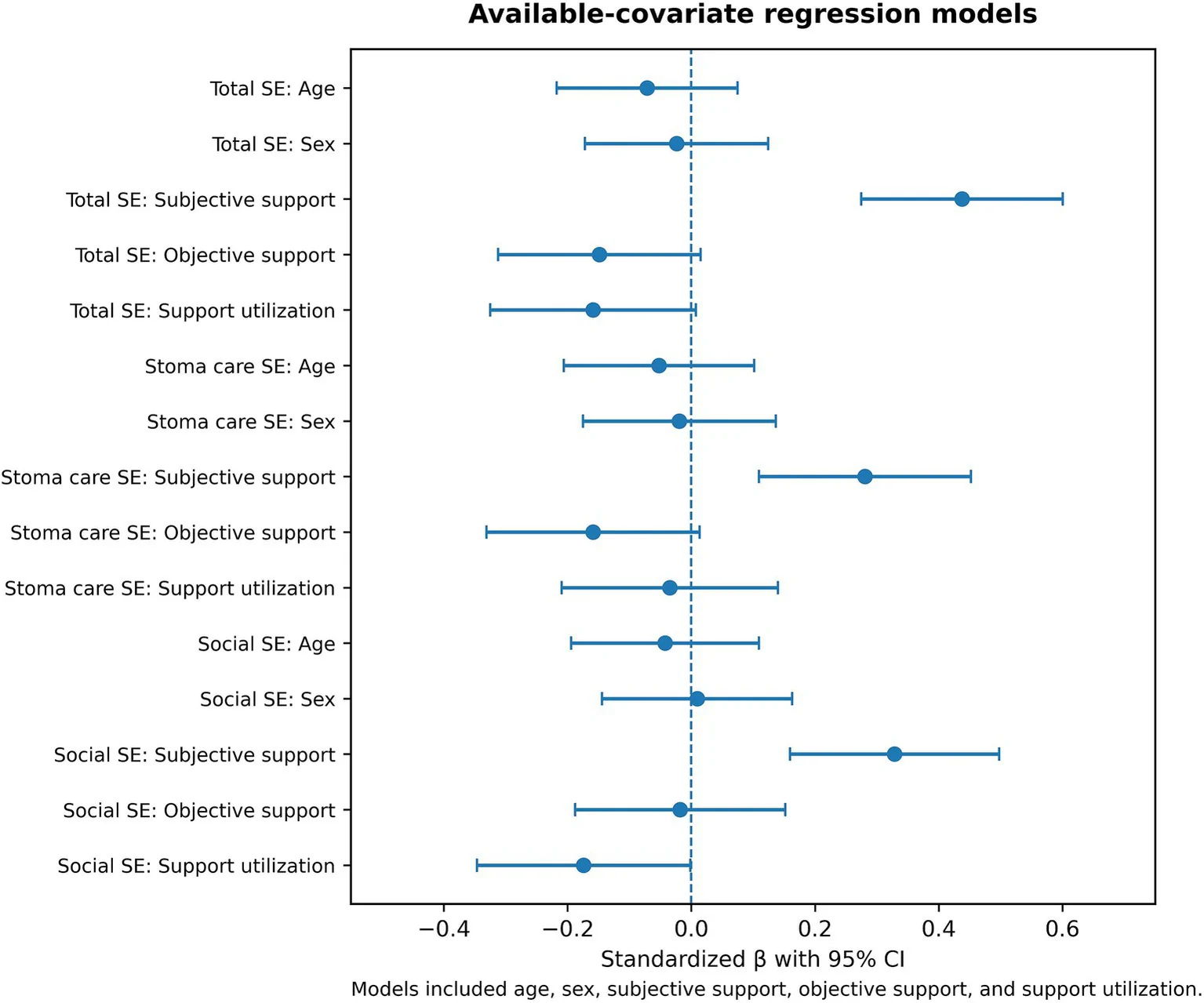
Distribution of standardized regression coefficients in exploratory available-covariate models for SSES-based self-efficacy outcomes. Standardized regression coefficients are shown for the multiple linear regression models for total self-efficacy, stoma care self-efficacy, and social self-efficacy. The dashed line indicates *β* = 0. Models included age, sex, subjective support, objective support, and support utilization.

The available-covariate model for total self-efficacy included age, sex, subjective support, objective support, and support utilization. The model was statistically significant (*R*^2^ = 0.159, adjusted *R*^2^ = 0.132, *F* = 5.904, *p* < 0.001). Subjective support was positively associated with total self-efficacy (*B* = 1.790, *β* = 0.438, *p* < 0.001). Objective support and support utilization did not reach the conventional significance threshold in the primary model (*p* = 0.075 and *p* = 0.061, respectively) (Table 7 and Figure 3).

**Table 7:** Exploratory multiple linear regression for SSES-based total self-efficacy using available covariates.

Variable	*B*	SE	*β*	*t*	*p-value*	95% CI	VIF
Intercept	78.356	8.137		9.630	<0.001	62.283, 94.429	
Age	−0.094	0.098	−0.071	−0.962	0.338	−0.287, 0.099	1.016
Sex (male vs. female)	−0.422	1.332	−0.024	−0.317	0.752	−3.054, 2.209	1.042
Subjective support	1.790	0.338	0.438	5.303	<0.001	1.123, 2.457	1.264
Objective support	−0.479	0.267	−0.148	−1.790	0.075	−1.007, 0.050	1.274
Support utilization	−0.919	0.487	−0.159	−1.888	0.061	−1.881, 0.043	1.313

Model included age, sex, subjective support, objective support, and support utilization because the available dataset did not contain the additional clinical and sociodemographic covariates requested by the reviewer. B = unstandardized coefficient; β = standardized coefficient; CI = confidence interval; VIF = variance inflation factor. Model summary: *N* = 162, *R*^2^ = 0.159, adjusted *R*^2^ = 0.132, *F* = 5.904, model *p* ≤ 0.001.

The available-covariate model for stoma care self-efficacy was statistically significant (*R*^2^ = 0.069, adjusted *R*^2^ = 0.040, *F* = 2.329, *p* = 0.045). Subjective support was positively associated with stoma care self-efficacy (*B* = 0.819, *β* = 0.281, *p* = 0.001). Age, sex, objective support, and support utilization were not statistically significant in this model (Table 8 and Figure 3).

**Table 8:** Exploratory multiple linear regression for SSES-based stoma care self-efficacy using available covariates.

Variable	*B*	SE	*β*	*t*	*p-value*	95% CI	VIF
Intercept	40.593	6.101		6.654	<0.001	28.542, 52.644	
Age	−0.049	0.073	−0.052	−0.669	0.505	−0.194, 0.096	1.016
Sex (male vs. female)	−0.242	0.999	−0.019	−0.242	0.809	−2.215, 1.731	1.042
Subjective support	0.819	0.253	0.281	3.235	0.001	0.319, 1.319	1.264
Objective support	−0.365	0.201	−0.159	−1.820	0.071	−0.761, 0.031	1.274
Support utilization	−0.143	0.365	−0.035	−0.391	0.696	−0.864, 0.578	1.313

Model included age, sex, subjective support, objective support, and support utilization because the available dataset did not contain the additional clinical and sociodemographic covariates requested by the reviewer. B = unstandardized coefficient; β = standardized coefficient; CI = confidence interval; VIF = variance inflation factor. Model summary: *N* = 162, *R*^2^ = 0.069, adjusted *R*^2^ = 0.040, *F* = 2.329, model *p* = 0.045.

The available-covariate model for social self-efficacy was statistically significant (*R*^2^ = 0.095, adjusted *R*^2^ = 0.066, *F* = 3.279, *p* = 0.008). Subjective support was positively associated with social self-efficacy (*B* = 0.658, *β* = 0.329, *p* < 0.001). Support utilization was negatively associated with social self-efficacy (*B* = −0.493, *β* = −0.174, *p* = 0.048). Age, sex, and objective support were not statistically significant. Model diagnostics showed maximum VIF = 1.313, Durbin-Watson values of 1.858–1.942, and non-significant Breusch-Pagan tests. Residual normality was not met in the total self-efficacy and stoma care self-efficacy models. HC3 robust standard errors retained positive associations for subjective support in all three outcomes (Supplementary Tables S1, S2, Table 9, and Figure 3).

**Table 9:** Exploratory multiple linear regression for SSES-based social self-efficacy using available covariates.

Variable	B	SE	*β*	*t*	*p-value*	95% CI	VIF
Intercept	23.566	4.132		5.704	<0.001	15.405, 31.728	
Age	−0.027	0.050	−0.042	−0.550	0.583	−0.125, 0.071	1.016
Sex (male vs. female)	0.082	0.676	0.009	0.122	0.903	−1.254, 1.419	1.042
Subjective support	0.658	0.171	0.329	3.838	<0.001	0.319, 0.997	1.264
Objective support	−0.028	0.136	−0.018	−0.209	0.835	−0.297, 0.240	1.274
Support utilization	−0.493	0.247	−0.174	−1.993	0.048	−0.981, −0.004	1.313

Model included age, sex, subjective support, objective support, and support utilization because the available dataset did not contain the additional clinical and sociodemographic covariates requested by the reviewer. B = unstandardized coefficient; *β* = standardized coefficient; CI = confidence interval; VIF = variance inflation factor. Model summary: *N* = 162, *R*^2^ = 0.095, adjusted *R*^2^ = 0.066, *F* = 3.279, model *p* = 0.008.

### Sensitivity analysis of subjective support stratification

**Figure 4:**
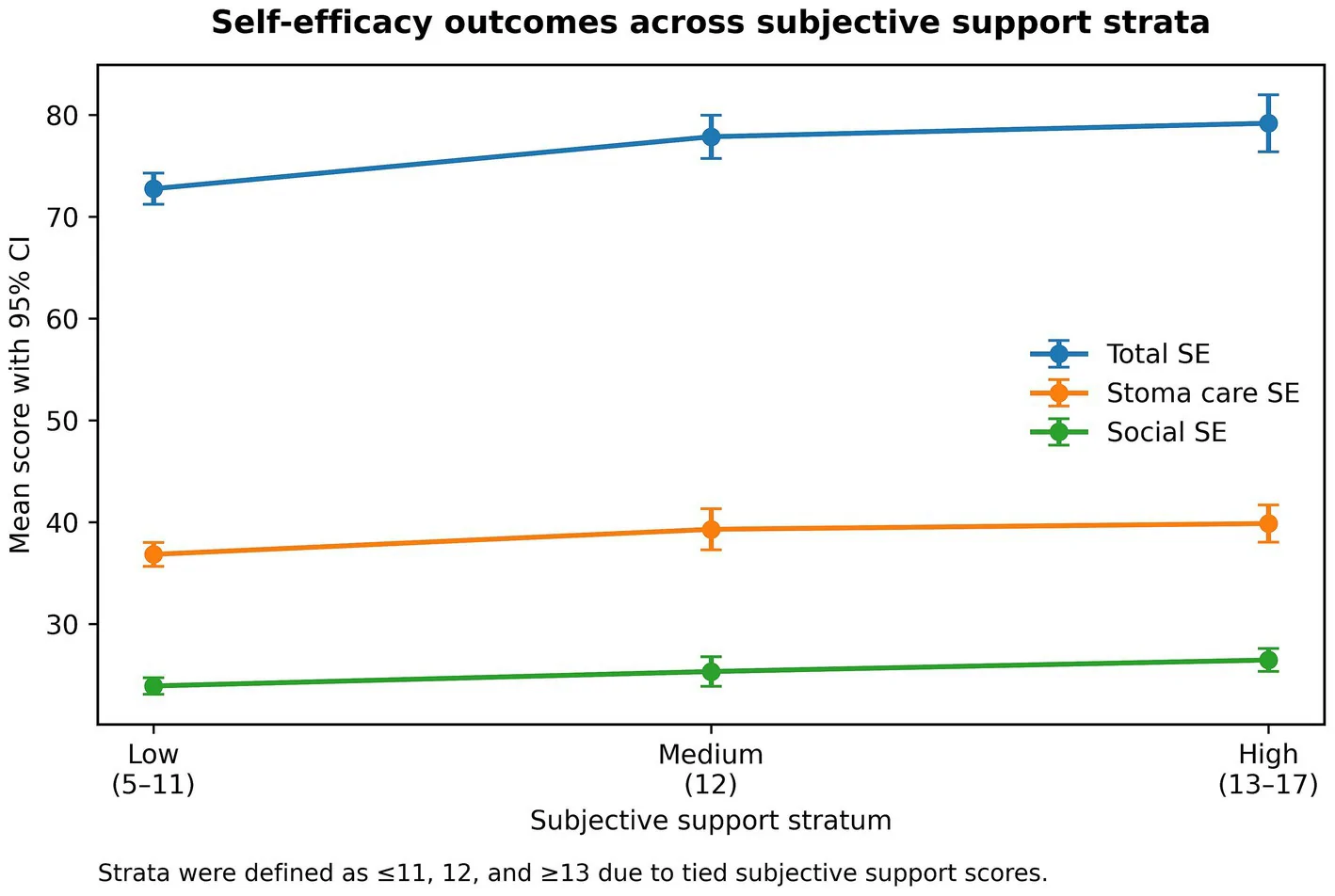
Mean scores of SSES-based total self-efficacy, stoma care self-efficacy, and social self-efficacy across subjective support strata.

Patients were categorized into low subjective support (5–11), medium subjective support (12), and high subjective support (13–17) groups based on sample tertiles with tied scores. Total self-efficacy scores differed across the three groups: 72.73 ± 6.98, 77.84 ± 5.90, and 79.17 ± 9.50, respectively (*p* < 0.001, η^2^ = 0.135). Stoma care self-efficacy scores were 36.82 ± 5.32, 39.28 ± 5.59, and 39.85 ± 6.26 (*p* = 0.007, η^2^ = 0.060). Social self-efficacy scores were 23.89 ± 3.79, 25.31 ± 4.00, and 26.45 ± 3.84 (*p* = 0.001, η^2^ = 0.079) (Table 10 and Figure 4).

**Table 10:** Exploratory sensitivity analysis of SSES-based self-efficacy outcomes by subjective support strata.

Outcome	Subjective support stratum	*n*	Mean	SD	*F*	ANOVA *p*-value	*P* for trend
Total self-efficacy	Low subjective support (5–11)	83	72.73	6.98	12.417	<0.001	<0.001
Total self-efficacy	Medium subjective support (12–12)	32	77.84	5.90	12.417	<0.001	<0.001
Total self-efficacy	High subjective support (13–17)	47	79.17	9.50	12.417	<0.001	<0.001
Stoma care self-efficacy	Low subjective support (5–11)	83	36.82	5.32	5.050	0.007	0.003
Stoma care self-efficacy	Medium subjective support (12–12)	32	39.28	5.59	5.050	0.007	0.003
Stoma care self-efficacy	High subjective support (13–17)	47	39.85	6.26	5.050	0.007	0.003
Social self-efficacy	Low subjective support (5–11)	83	23.89	3.79	6.830	0.001	<0.001
Social self-efficacy	Medium subjective support (12–12)	32	25.31	4.00	6.830	0.001	<0.001
Social self-efficacy	High subjective support (13–17)	47	26.45	3.84	6.830	0.001	<0.001

Subjective support strata were defined using tertiles of the current sample distribution. Because of tied scores, strata corresponded to scores ≤11, 12, and ≥13. P for trend was obtained by treating strata as an ordinal variable.

Trend tests across subjective support strata were statistically significant for total self-efficacy (*p* for trend < 0.001), stoma care self-efficacy (*p* for trend = 0.003), and social self-efficacy (*p* for trend < 0.001). This sensitivity analysis was treated as a trend across subjective support strata (Table 10 and Figure 4).

### Stratified analysis of the relationship between subjective support and self-efficacy across different subgroups

Sex-stratified analyses were exploratory. Subjective support was associated with total self-efficacy in males (*B* = 1.537, 95% CI: 0.754 to 2.320, *p* < 0.001), while the association in females did not reach statistical significance (*B* = 0.731, 95% CI: −0.150 to 1.611, *p* = 0.102). Similar descriptive patterns were observed for stoma care self-efficacy and social self-efficacy (Table 11).

**Table 11:** Exploratory subgroup analyses of the association between subjective support and SSES-based self-efficacy outcomes.

Outcome	Stratification variable	Subgroup	*n*	Subjective support B	SE	95% CI	*p-*value	*R* ^2^
Total self-efficacy	Sex	Male	114	1.537	0.395	0.754, 2.320	<0.001	0.119
Total self-efficacy	Sex	Female	48	0.731	0.437	−0.150, 1.611	0.102	0.057
Total self-efficacy	Age group	60–69 years	126	1.448	0.366	0.723, 2.174	<0.001	0.112
Total self-efficacy	Age group	70–79 years	27	1.060	0.612	−0.201, 2.321	0.096	0.107
Total self-efficacy	Age group	≥80 years	9	1.350	1.482	−2.155, 4.855	0.393	0.106
Stoma care self-efficacy	Sex	Male	114	0.723	0.280	0.167, 1.279	0.011	0.056
Stoma care self-efficacy	Sex	Female	48	0.286	0.376	−0.471, 1.043	0.451	0.012
Stoma care self-efficacy	Age group	60–69 years	126	0.650	0.267	0.121, 1.178	0.016	0.046
Stoma care self-efficacy	Age group	70–79 years	27	0.540	0.437	−0.360, 1.440	0.228	0.058
Stoma care self-efficacy	Age group	≥80 years	9	0.750	1.490	−2.773, 4.273	0.630	0.035
Social self-efficacy	Sex	Male	114	0.570	0.190	0.194, 0.945	0.003	0.075
Social self-efficacy	Sex	Female	48	0.369	0.259	−0.153, 0.892	0.161	0.042
Social self-efficacy	Age group	60–69 years	126	0.629	0.183	0.267, 0.992	<0.001	0.087
Social self-efficacy	Age group	70–79 years	27	0.284	0.284	−0.301, 0.869	0.327	0.038
Social self-efficacy	Age group	≥80 years	9	0.300	0.863	−1.740, 2.340	0.738	0.017

Subgroup analyses were exploratory and used unadjusted within-subgroup linear regression models. Small subgroups, especially the ≥80 years group, should not be used to infer stable effect modification.

Age-stratified analyses were also exploratory. Subjective support was associated with total self-efficacy in the 60–69-year group (*B* = 1.448, 95% CI: 0.723 to 2.174, *p* < 0.001), while associations in the 70–79-year group and ≥80-year group did not reach statistical significance. The ≥80-year group included 9 patients. Formal interaction tests for subjective support by sex and age group were not statistically significant after FDR correction for total self-efficacy, stoma care self-efficacy, or social self-efficacy (all q > 0.05). No robust evidence of effect modification by sex or age group was observed (Table 11 and Supplementary Table S3).

## Discussion

This cross-sectional study examined social support and self-efficacy among older adults 1–3 months after first intestinal stoma surgery. Social support and self-efficacy scores were described as continuous outcomes. The sample had higher objective support scores than subjective support and support utilization scores. Stoma care self-efficacy scores were higher than social self-efficacy scores. These patterns are consistent with studies reporting moderate social support and postoperative adaptation among patients with intestinal stoma (Wei et al., 2025; Martin-Gil et al., 2024). Studies in Guangxi older adults with intestinal stoma have also reported moderate social support, with clinical and psychosocial correlates (Wei et al., 2025).

A critical consideration in interpreting these findings is the limited internal consistency of the SSES in this sample. Although the scoring procedure and item coding were rechecked, Cronbach’s alpha remained low for the total scale and especially for the social self-efficacy dimension. This finding contrasts with previous validation studies, in which the original stoma-care and social self-efficacy subscales showed high internal consistency (Cronbach’s alpha = 0.94 and 0.95, respectively), the Turkish validation study reported Cronbach’s alpha values of 0.95 for the total scale, 0.92 for stoma-care self-efficacy, and 0.93 for social self-efficacy, and the Chinese version cited in the validation literature showed high subscale reliability. The recent Czech validation study also reported high internal consistency for the total 28-item SSES and strong reliability across extracted factors, although it identified a three-factor structure that separated stoma care, social, and burden-related self-efficacy domains (Machalkova et al., 2025). The discrepancy suggests that the present coefficients are more likely to reflect sample-specific measurement instability, clinical-context heterogeneity, or limited applicability of some SSES items in older adults 1–3 months after surgery, rather than the expected reliability of the instrument across all settings. The SSES was therefore retained because it is a stoma-specific self-efficacy instrument and because coding and aggregation were verified, but all SSES-based analyses were interpreted as exploratory and reliability-limited. The observed associations between subjective support and self-efficacy should not be regarded as definitive evidence based on a highly reliable outcome measure.

Sexual activity and sexual satisfaction confidence had lower item scores than most other self-efficacy items. Prior studies have described body image concerns, sexual health concerns, and unmet support needs among individuals living with intestinal stoma (Fourie et al., 2025; Redeker et al., 2025). Literature reviews have also reported effects of intestinal stoma on body image, partner relationships, and sexual quality of life (Wang et al., 2024). In this study, these domains were retained as descriptive item-level findings. They were not used as independent mechanistic endpoints.

Self-efficacy has also been examined in relation to stoma adaptation and self-management in patients with intestinal stoma. A previous study reported an association between self-efficacy levels and stoma adaptation in patients with intestinal stoma (Ozden and Kilic, 2023). A path analysis in colorectal cancer patients with a stoma reported links among social support, self-efficacy, and self-management ability (Xu et al., 2024). These studies provide a comparative context for the present findings. The present sample was restricted to older adults 1–3 months after first intestinal stoma surgery, and the results should be interpreted within this postoperative time window.

Total social support was not correlated with total self-efficacy or its main dimensions after FDR correction. Subjective support was correlated with total self-efficacy and social self-efficacy after FDR correction. This pattern is compatible with prior studies reporting associations between perceived social support and stoma self-efficacy (Aker and Karazeybek, 2025; Xu et al., 2024). Total social support combines objective support, subjective support, and support utilization. Dimension-specific analyses may yield different correlation patterns than the total score. This interpretation remains descriptive because all exposure and outcome variables were measured at the same time.

The difference between total social support and subjective support should be interpreted cautiously. The SSRS total score combines objective support, subjective support, and support utilization. Associations with the total score may be attenuated when component dimensions have different directions and magnitudes. Subjective support and self-efficacy were both measured by self-report scales. Their correlation may partly reflect shared perceptual and emotional variance, including general psychological adjustment, positive affect, and optimism. The cross-sectional design cannot separate a dimension-specific association from common method variance.

Intervention studies provide background for future research, not causal evidence for the present findings. Home videoconferencing education has been reported to increase self-efficacy and stoma adaptation in individuals with a stoma (Ozkaya and Harputlu, 2024). Post-discharge health education has also been associated with higher self-care ability and psychosocial adaptation in enterostomy patients (Ko et al., 2023). In the present study, subjective support was assessed as a psychosocial exposure dimension rather than an intervention target. Future longitudinal and intervention studies should collect clinical covariates, psychosocial covariates, and non-self-report measures to examine whether changes in perceived support precede changes in self-efficacy trajectories.

Several limitations should be considered. First, the cross-sectional design does not establish temporal order or causality. Second, only age and sex were available as non-scale covariates in the analytic dataset. Stoma type, primary diagnosis, postoperative complications, time since surgery, educational level, marital status, living situation, number of comorbidities, psychological distress, functional dependence, and prior self-care experience were not systematically available. Residual confounding remains possible. Third, SSRS and SSES are self-report scales. Shared perceptual or emotional variance, response bias, and social desirability bias may have affected the associations. Fourth, the SSES showed low internal consistency in this cohort despite verification of item coding and score aggregation. This reliability limitation means that SSES-based estimates may be attenuated, unstable, or difficult to interpret as precise measurements of stoma-related self-efficacy, and the findings should be regarded as exploratory associations requiring confirmation with psychometrically robust measurement. Fifth, the regression models explained modest proportions of variance, with R^2^ values of 0.159, 0.069, and 0.095. Sixth, this was a single-center study from a tertiary hospital in Nanning, Guangxi. The sample was concentrated in males and patients aged 60–69 years. Generalizability to younger patients, long-term intestinal stoma patients, female-predominant samples, non-tertiary settings, other regions, and other sociocultural contexts is limited. Future studies should use multicenter longitudinal designs, collect clinical and psychosocial covariates, include non-self-report measures, and evaluate whether support-focused interventions alter self-efficacy trajectories after intestinal stoma surgery.

The main limitation of this study is that the SSES showed low internal consistency in the current cohort despite verification of item coding and reliability calculation and despite prior validation studies reporting substantially higher reliability. This limitation may attenuate or destabilize associations involving self-efficacy outcomes and reduces confidence in precise effect-size interpretation. The cross-sectional, single-center design and the absence of broader clinical and sociodemographic covariates further limit causal inference and generalizability.

## Conclusion

Among older adults 1–3 months after first intestinal stoma surgery, subjective support was associated with SSES-based self-efficacy scores after FDR correction and in available-covariate regression models, with modest model explanatory power. Total social support, objective support, and support utilization were not consistently associated with the main self-efficacy outcomes after FDR correction. Because the SSES demonstrated limited internal consistency in this cohort, in contrast to higher reliability reported in previous validation studies, these findings should be interpreted cautiously as exploratory and reliability-limited associations requiring confirmation in larger studies using psychometrically robust self-efficacy measurement. The cross-sectional design, residual confounding, self-report measurement, and single-center sample preclude causal interpretation.

## Data Availability

The original contributions presented in the study are included in the article/Supplementary material, further inquiries can be directed to the corresponding author/s.

## Supplementary material

SUPPLEMENTARY FIGURE S1 Flow chart of study subject inclusion.
