# Supplementary material for: Perceived subjective support is associated with self-efficacy in older adults early after intestinal stoma surgery: a cross-sectional study with FDR correction
Source: Front Psychol. 2026 Aug 7;17:1850651. doi: 10.3389/fpsyg.2026.1850651 (PMC13493508; doi:10.3389/fpsyg.2026.1850651)
Supplement: Supplementary file 1 [file Supplementary_file_1.DOCX]

**Table S1. Residual diagnostic statistics for the available-covariate regression models.**

| **Outcome** | **Shapiro-Wilk P** | **Jarque-Bera P** | **Breusch-Pagan P** | **Durbin-Watson** | **Maximum VIF** | **Cook’s distance > 4/n** | **Maximum Cook’s distance** |
| --- | --- | --- | --- | --- | --- | --- | --- |
| Total self-efficacy | <0.001 | <0.001 | 0.639 | 1.858 | 1.313 | 7 | 0.104 |
| Stoma care self-efficacy | 0.002 | <0.001 | 0.469 | 1.864 | 1.313 | 10 | 0.055 |
| Social self-efficacy | 0.241 | 0.210 | 0.563 | 1.942 | 1.313 | 10 | 0.124 |

Note: The same available-covariate models as Tables 7-9 were evaluated. Breusch-Pagan *p value*s assess heteroscedasticity. Durbin-Watson values near 2 indicate no strong residual autocorrelation.

**Table S2. HC3 robust standard error sensitivity analysis for the available-covariate regression models.**

| **Outcome** | **Variable** | **B** | **Robust SE (HC3)** | **Robust *p value*** | **Robust 95% CI** |
| --- | --- | --- | --- | --- | --- |
| Total self-efficacy | Intercept | 78.356 | 8.006 | <0.001 | 62.664, 94.048 |
|  | Age | -0.094 | 0.090 | 0.294 | -0.270, 0.082 |
|  | Sex (male vs female) | -0.422 | 1.165 | 0.717 | -2.705, 1.861 |
|  | Subjective support | 1.790 | 0.283 | <0.001 | 1.236, 2.344 |
|  | Objective support | -0.479 | 0.244 | 0.049 | -0.956, -0.001 |
|  | Support utilization | -0.919 | 0.441 | 0.037 | -1.784, -0.055 |
| Stoma care self-efficacy | Intercept | 40.593 | 6.284 | <0.001 | 28.276, 52.910 |
|  | Age | -0.049 | 0.074 | 0.507 | -0.194, 0.096 |
|  | Sex (male vs female) | -0.242 | 0.952 | 0.799 | -2.109, 1.624 |
|  | Subjective support | 0.819 | 0.205 | <0.001 | 0.416, 1.221 |
|  | Objective support | -0.365 | 0.209 | 0.080 | -0.774, 0.044 |
|  | Support utilization | -0.143 | 0.362 | 0.693 | -0.852, 0.566 |
| Social self-efficacy | Intercept | 23.566 | 4.648 | <0.001 | 14.457, 32.676 |
|  | Age | -0.027 | 0.051 | 0.590 | -0.127, 0.072 |
|  | Sex (male vs female) | 0.082 | 0.656 | 0.900 | -1.204, 1.368 |
|  | Subjective support | 0.658 | 0.175 | <0.001 | 0.315, 1.001 |
|  | Objective support | -0.028 | 0.145 | 0.844 | -0.312, 0.255 |
|  | Support utilization | -0.493 | 0.250 | 0.049 | -0.983, -0.003 |

Note: HC3 robust standard errors were used as a sensitivity analysis because residual normality was not fully satisfied in some models.

**Table S3. Formal interaction tests for subjective support by sex and age group.**

| **Outcome** | **Interaction term** | **df added** | **F** | ***p value*** | **q value** | **Coefficient/description** |
| --- | --- | --- | --- | --- | --- | --- |
| Total self-efficacy | Subjective support × sex | 1 | 1.824 | 0.179 | 0.736 | B = 0.905 (-0.419, 2.230) |
| Total self-efficacy | Subjective support × age group | 2 | 0.316 | 0.730 | 0.876 | Overall interaction test |
| Stoma care self-efficacy | Subjective support × sex | 1 | 0.865 | 0.354 | 0.736 | B = 0.469 (-0.527, 1.465) |
| Stoma care self-efficacy | Subjective support × age group | 2 | 0.073 | 0.929 | 0.929 | Overall interaction test |
| Social self-efficacy | Subjective support × sex | 1 | 0.515 | 0.474 | 0.736 | B = 0.245 (-0.430, 0.921) |
| Social self-efficacy | Subjective support × age group | 2 | 0.715 | 0.491 | 0.736 | Overall interaction test |

Note: q values were obtained using Benjamini–Hochberg false discovery rate correction across the six interaction tests. No interaction remained significant after correction.

**Table S4. Additional reliability diagnostics for the Stoma Self-Efficacy Scale (SSES).**

| **Scale or dimension** | **Items** | **N** | **Missing item cells** | **Cronbach's alpha** | **Mean inter-item r** | **Median inter-item r** | **Corrected item-total r range** | **Alpha-if-item-deleted range** | **Maximum floor effect (%)** | **Maximum ceiling effect (%)** |
| --- | --- | --- | --- | --- | --- | --- | --- | --- | --- | --- |
| Total SSES | 28 | 162 | 0 | 0.378 | 0.022 | 0.02 | -0.103 to 0.216 | 0.341 to 0.406 | 71.6 | 26.5 |
| Stoma care self-efficacy dimension | 13 | 162 | 0 | 0.324 | 0.037 | 0.039 | -0.014 to 0.238 | 0.257 to 0.351 | 19.8 | 26.5 |
| Social self-efficacy dimension | 9 | 162 | 0 | 0.019 | 0.002 | 0.016 | -0.093 to 0.083 | -0.045 to 0.095 | 22.2 | 20.4 |
| Six individual SSES items | 6 | 162 | 0 | 0.1 | 0.02 | 0.018 | -0.038 to 0.103 | 0.005 to 0.172 | 71.6 | 3.1 |

Note. The table summarizes item-level diagnostics without displaying the full item correlation matrix. These results support treating SSES-based findings as exploratory and reliability-limited in this cohort.
